# Case report: Myelitis and ganglionitis, an atypical presentation of Hansen’s disease

**DOI:** 10.3389/fmed.2024.1400423

**Published:** 2024-05-20

**Authors:** Clarissa Neves Spitz, Izabela Jardim R. Pitta, Ligia Andrade, Larissa Bittencourt de Carvalho, Diogo Matheus Terrana de Carvalho, Felipe da Rocha Schmidt, Ana Caroline Siquara de Sousa, Silvana Machado Mendonça, Euzenir Nunes Sarno, Anna Maria Sales, Roberta Olmo Pinheiro, Marcia Rodrigues Jardim

**Affiliations:** ^1^Post-Graduate Program in Neurology, Federal University of the State of Rio de Janeiro, Rio de Janeiro, Brazil; ^2^Laboratory of Leprosy, Oswaldo Cruz Institute, Oswaldo Cruz Foundation (FIOCRUZ), Rio de Janeiro, Rio de Janeiro, Brazil; ^3^Pedro Ernesto University Hospital, Rio de Janeiro State University, Rio de Janeiro, Brazil; ^4^Department of Neurology, Fluminense Federal University, Niterói, Rio de Janeiro, Brazil; ^5^Clinical Diagnostic Imaging (CDPI), Rio de Janeiro, Rio de Janeiro, Brazil; ^6^National Institute of Science and Technology on Neuroimmunomodulation, Rio de Janeiro, Rio de Janeiro, Brazil

**Keywords:** Hansen’s disease, atypical, central nervous system, resonance magnetic images, ultrasound imaging, ulnar nerve

## Abstract

Hansen’s disease, or leprosy, is a disease characterized by dermatological and neurological disorders. A neural form also exists, in which peripheral neuropathy occurs in the absence of skin lesions. However, cases of leprosy that involve the central nervous system and proximal nerves are rare in the literature. We describe the case of an oligosymptomatic patient diagnosed with the neural form of leprosy with involvement of peripheral nerves, dorsal root ganglion, and cervical spinal cord in an atypical presentation of the disease. Through complementary examinations and nerve biopsies, the bacillus was identified, and treatment was subsequently initiated. This case highlights the importance of investigating the suspicion of leprosy, even in cases with atypical manifestations, as early diagnosis and treatment can reduce neurological damage and deformities.

## Introduction

Hansen’s disease, also known as leprosy, is a disease characterized by dermatological and neurological disorders. Early diagnosis and treatment are crucial to reducing the impact of neurological impairment, which can cause deformities and physical disabilities (1). Leprosy neuropathy can be acute or chronic, and neural involvement can occur before, during, or after multidrug therapy (1). Depending on an individual’s response to *Mycobacterium leprae*, the disease progresses, often resulting in the thickening of the nerve trunks.

Neuritis is characterized by pain that can occur spontaneously or upon palpation, often in conjunction with sensory impairment. It may also include signs of motor impairment in the corresponding nerve. Nerve enlargement is common in neuritis, and nerve conduction studies can help support the diagnosis when signs of demyelination are present (2–4). Neuritis strictly means inflammation of the nerve, implying involvement of the host’s system. Neuritis can be seen in type 1 and type 2 leprosy reactions and as isolated neuritis without skin lesions (5–8). Neuritis with painless impairment of nerve function can also occur, which may or may not be associated with cutaneous reaction episodes (9).

A neural form of leprosy also exists, which manifests as peripheral neuropathy in the absence of skin lesions (10, 11). This is a rare presentation of the disease, and thus, its diagnosis and management are a challenge (11). At the Souza Araújo Outpatient Clinic, a Leprosy Reference Center at the Oswaldo Cruz Institute in Rio de Janeiro, Brazil, 7.8% of patients with leprosy were diagnosed with the neural form between 1998 and 2016 (12). The definitive diagnosis can be achieved by performing a peripheral nerve biopsy (13, 14). Electroneuromyography helps to localize the nerve eligible for biopsy, with axonal involvement being the most frequent electrophysiological finding. The presence of acid-fast bacilli, epithelioid granulomas, carrier macrophages, and caseous necrosis in the nerve biopsy, as well as the detection of *M. leprae* DNA via the polymerase chain reaction (PCR), define the diagnosis of the neural form (15).

Cases of leprosy involving the central nervous system (CNS) and proximal nerves are scarcely reported in the literature (16–19). Although limited, radiological findings may have implications for understanding the pathophysiology of the disease and therapeutic considerations in leprosy. In this case, we describe an oligosymptomatic patient diagnosed with the neural form of leprosy with the involvement of peripheral nerves, dorsal root ganglion, and cervical spinal cord in a completely atypical presentation of the disease.

## Case description

A 35-year-old man presented with progressive paresthesia on the medial surface of the right forearm and hand, which began 5 months before he visited our clinic. After 1 month, the patient noticed hypoesthesia in the same region associated with paresis and abduction of the fourth and fifth fingers on the right hand. During the same period, the patient noticed a painless nodulation in the proximal region of the medial epicondyle of the upper right limb; however, pain occurred upon palpation. An investigation was carried out at the primary care level, with suspected ulnar nerve involvement, and the patient was subsequently referred to our clinic, the Souza Araújo Outpatient Clinic, for evaluation. The patient did not report any allergies or regular use of medication, but he had undergone resection of a benign tumor (oncocytoma) in the right parotid region 1 and a half years prior.

A dermatological evaluation was performed, which was normal, and routine bacilloscopy of slit-skin smears was negative. Upon neurological examination, thickening of the right ulnar nerve was detected above the elbow. Paresthesia and shock-like pain in the right ulnar nerve were reported to worsen with touch (allodynia). Brisk deep tendon reflexes globally (3+). Vibratory, discriminative sensitivity, and proprioception were preserved; however, tactile sensitivity was reduced, and thermal and pain sensitivities were abolished in the right ulnar nerve territory. Hypoesthesia was present in the medial cutaneous nerve territory of the right forearm. Strength was reduced to grade 3 on the Medical Research Council (MRC) scale (20) in the right first dorsal interosseous and abductor digiti minimi muscles. Cranial nerves were normal. The remainder of the neurological examination was normal.

In electroneuromyography, sensory action potentials were absent in the right ulnar nerve and medial cutaneous nerve of the forearm (MCF), and compound motor action potentials were absent in the right ulnar nerve with capture in the abductor digit minimi muscle. In other evaluated nerves, including those of the lower limbs, no alterations were observed. In myography, spontaneous potentials with a pattern of positive waves and fibrillations were observed in the right extensor digitorum communis, flexor carpi ulnaris, and first dorsal interosseus muscles. Recruitment of motor unit potentials was reduced in these muscles as well as in the abductor pollicis brevis.

Ultrasonography: A significant increase in the cross-sectional area (CSA) of the right ulnar and MCF nerves from the proximal level of the forearm to the axilla was observed in ultrasonography (USG). The brachial plexus did not exhibit any thickening. Maximum CSA measurements of 1.12 cm^3^ and 45 mm^3^ were determined for the right ulnar nerve and MCF nerves at the supra epicondylar level, respectively. Both nerves presented homogeneous fascicular dilatation with the loss of intraneural morphology and hypoechogenicity. Power Doppler showed the presence of flow. Other nerves were evaluated in the upper limbs without significant ultrasound abnormalities. Contralateral CSA values were 10 mm^3^ and 2 mm^3^ for the ulnar and MCF nerves, respectively.

Given the evident involvement of the ulnar and MCF nerves, along with USG signs of proximal involvement, the possibility of plexopathy was suggested. Therefore, the patient was referred for magnetic resonance imaging (MRI), which confirmed this suspicion (as described below).

**Figure 1:**
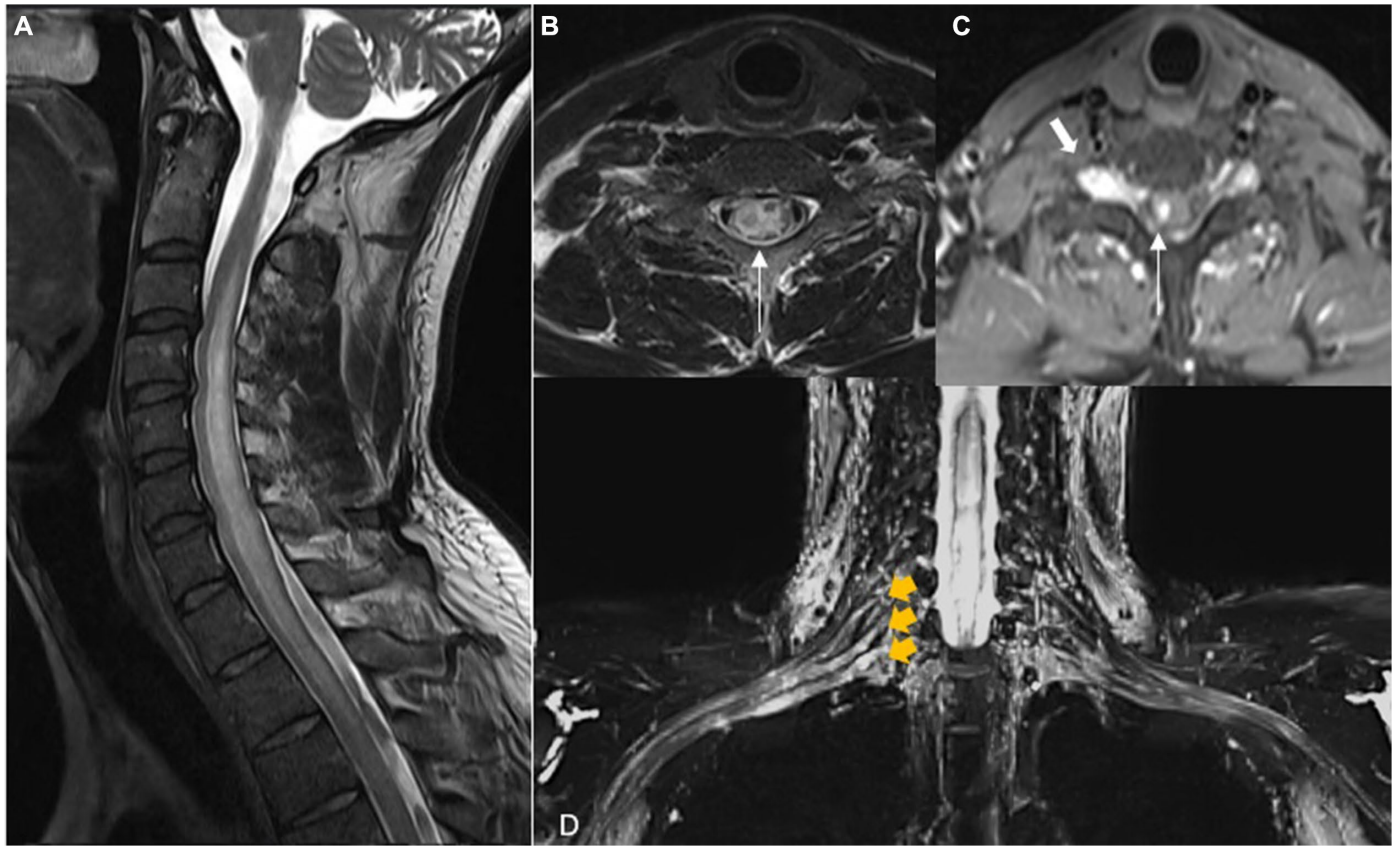
Magnetic resonance of the spine and brachial plexus. Sagittal **(A)** T2 shows extensive intramedullary lesion with high signal intensity and cord swelling from C2-T3. Axial T2 **(B)** and T1 FS **(C)** after gadolinium show the most evident increase in signal and swelling on the right side of the medulla and unilateral contrast enhancement (long arrows), suggesting a spinal inflammatory process. Note the enlargement of the root ganglion on the right side (short arrow). **(D)** Coronal STIR MIP is the irregular and extensive thickening and edema of C7, C8, and T1 roots and inferior and middle trunks of the right brachial plexus (yellow arrows).

**Figure 2:**
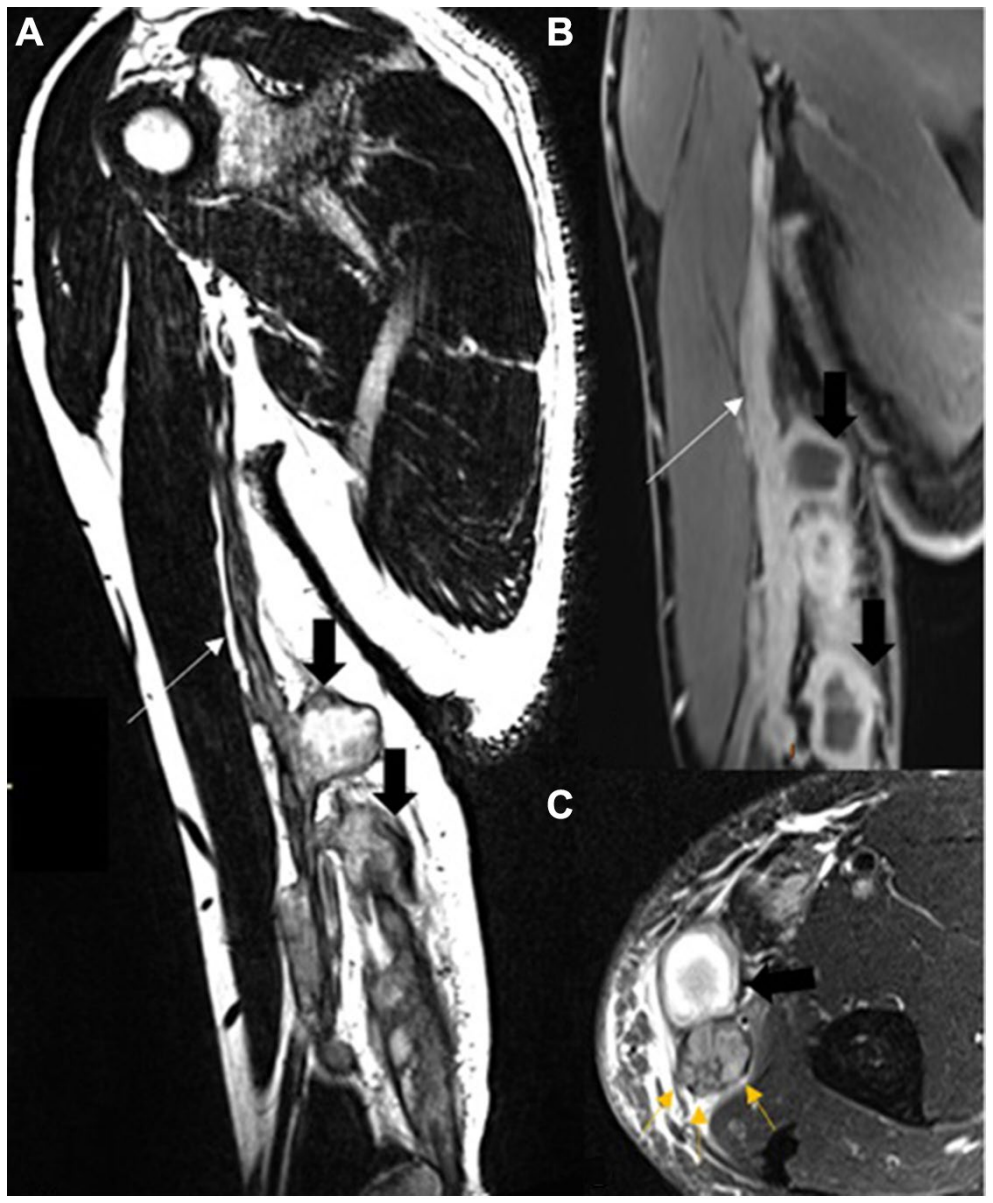
A magnetic resonance of the right arm coronal oblique T2 SPACE **(A)** and T1 FS after contrast **(B)**. Diffuse and lobulated ulnar and medial cutaneous nerve thickening and neural enhancement (long arrows) and large intraneural cavitations (short black arrows). Axial T2 FS **(C)**, the large cavitation (short black arrow), and neural thickening. Note the multiple fascicule enlargement at the non-cavitated portion (yellow arrows).

Magnetic resonance neurography of the cervical spine, brachial plexus, right arm, and elbow was performed. An extensive lesion with a hyperintense signal was observed on T2-weighted and short tau inversion recovery (STIR) images of the cervical spinal cord, which affected the right hemimedulla and exhibited an expansive effect, extending from the C2 vertebra to the level of D2–D3. An elongated/fusiform area was noticeable with contrast enhancement, extending from C6 to D1 and measuring approximately 3.8 × 0.6 × 0.5 cm (W × T × AP). The appearance suggested inflammatory change/myelitis. Edema, thickening, and enhancement of the neural ganglions of C6, C7, C8, and T1 on the right were also observed, associated with contrast enhancement compatible with ganglionitis. In neurography of the brachial plexus, marked thickening and contrast enhancement of the neural roots were evident in C7 and, notably, in C8 and T1, compatible with radiculitis. A marked increase in thickness with a heterogeneous edema signal was observed in the right ulnar and MCF nerves, from the axillary fold to the region of the proximal forearm. Heterogeneous fascicular thickening was noted, with a hyperintense signal on T1 and restricted diffusibility (thick content/granulomatous), and with multiple small lobulations and small hypointense punctate foci inside. The appearance was compatible with extensive diffuse neuropathies, including myelitis, ganglionitis, and radiculitis. Infectious or granulomatous inflammatory etiologies were considered the main hypotheses at this point. The images obtained in the pre- and post-treatment of the case are shown in Figures 1A–D, 2A–C.

The cerebrospinal fluid was clear and colorless, with a cellularity of 15 lymphocytes/μl (lymph/mono), 5 erythrocytes/μl, and protein and glucose levels of 68 mg/dL and 50 mg/dL, respectively. Serological tests, direct investigation for fungi, and cultures for fungi and bacteria were all negative. No neoplastic cells or IgG oligoclonal bands were detected, and the IgG index was normal.

More accurate examinations were performed to define the degree of neurological involvement, and other complementary examinations were conducted for etiological investigation. Positron emission tomography (PET) with 2-deoxy-2-[fluorine-18] fluoro-D-glucose (^18^F-FDG) revealed uptake in the right ulnar and MCF nerves. Computed tomography of the chest showed small streaks of fibrosis in the upper lobes, while the remaining lung parenchyma showed no significant changes. No evidence of lymph node enlargement or pleural effusion was observed, and brain and lumbar spine MRIs showed no significant changes.

Routine laboratory tests (biochemistry and blood counts) were normal, serological tests to detect infectious diseases were negative, and no abnormalities were observed in the levels of inflammatory markers (including the erythrocyte sedimentation rate and C-reactive protein test). Rheumatology screening was normal, and anti-ganglioside antibody and angiotensin-I-converting enzyme (ACE) tests were negative. Anti-myelin oligodendrocyte glycoprotein (MOG) and anti-aquaporin-4 antibody tests were also negative.

**Figure 3:**
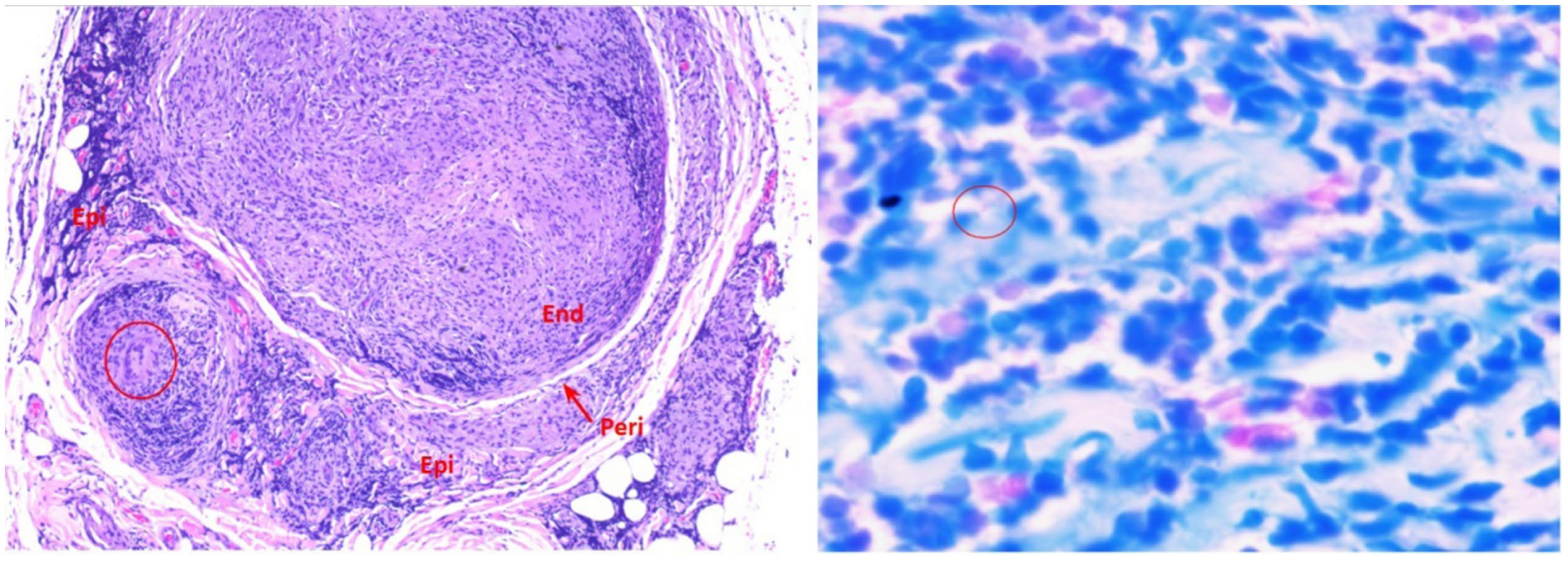
A microscopic image in hematoxylin and eosin, 100x. Presence of marked mononuclear inflammatory infiltrates around the vessels, fascicles, and in the fibroadipose tissue. The inflammatory infiltrate is also present in the perineurium and endoneurium, which shows outlines of granulomas. The circle highlights a giant Langhans cell. B Wade, 1,000x. Presence of moderate to marked mononuclear inflammatory infiltrate in the endoneurium and prominent bacilli (circle).

After extensive evaluation, a decision was made to perform a biopsy of the right dorsal cutaneous nerve, which yielded inconclusive results. Given the neurological manifestations and imaging findings, but without the etiology, the team decided to perform a new USG-guided biopsy of the right MCF. This biopsy revealed several histopathological changes. These changes included the presence of a mononuclear inflammatory infiltrate (with foamy macrophages) in the epineurium, perineurium, and endoneurium; fibrosis in the epineurium and endoneurium; the absence of myelin fibers (observed in semithin sections); and the presence of acid-fast bacilli in foamy macrophages (Wade-Fite stain, 1+/4+) (Figure 3). Upon comparing the histopathological findings of this nerve biopsy with the diagnostic criteria for biopsies of pure neural leprosy (15), it was classified as “category 1: confirmed diagnosis.” The criteria present include mononuclear inflammatory infiltrate in the nerve, fibrosis, a decreased number of myelin fibers, and the presence of AFB in the special stain (four of the nine histopathological changes most frequently found in the study cases). Because of the positive Wade-Fite staining, the case was included in this category. The histopathological images are shown in Figures 3A,B.

The diagnosis of an atypical form of leprosy was performed, represented by the involvement of peripheral nerves and the CNS in the form of myelitis and ganglionitis. Furthermore, the case had neural involvement without dermatological lesions, constituting the neural form of the disease. Multidrug therapy (12 monthly doses) was started as well as a pulse therapy regimen of corticosteroids (8 monthly doses) owing to the intense neurological involvement and inflammatory response.

The patient was evaluated monthly by the dermatology and neurology teams throughout the treatment, and USG and MRI were carried out in the 6th and 12th months of treatment. The patient underwent a pulse therapy regimen of weekly corticosteroids, spaced monthly, totaling eight applications. This condition was necessary due to the intense neurological damage and the inflammatory response observed. An immunosuppressant (azathioprine) was started to reduce the use of corticosteroids and was administered as a maintenance treatment. The patient adapted well to the regimen, remaining without corticosteroids and receiving an optimized dose of azathioprine.

**Figure 4:**
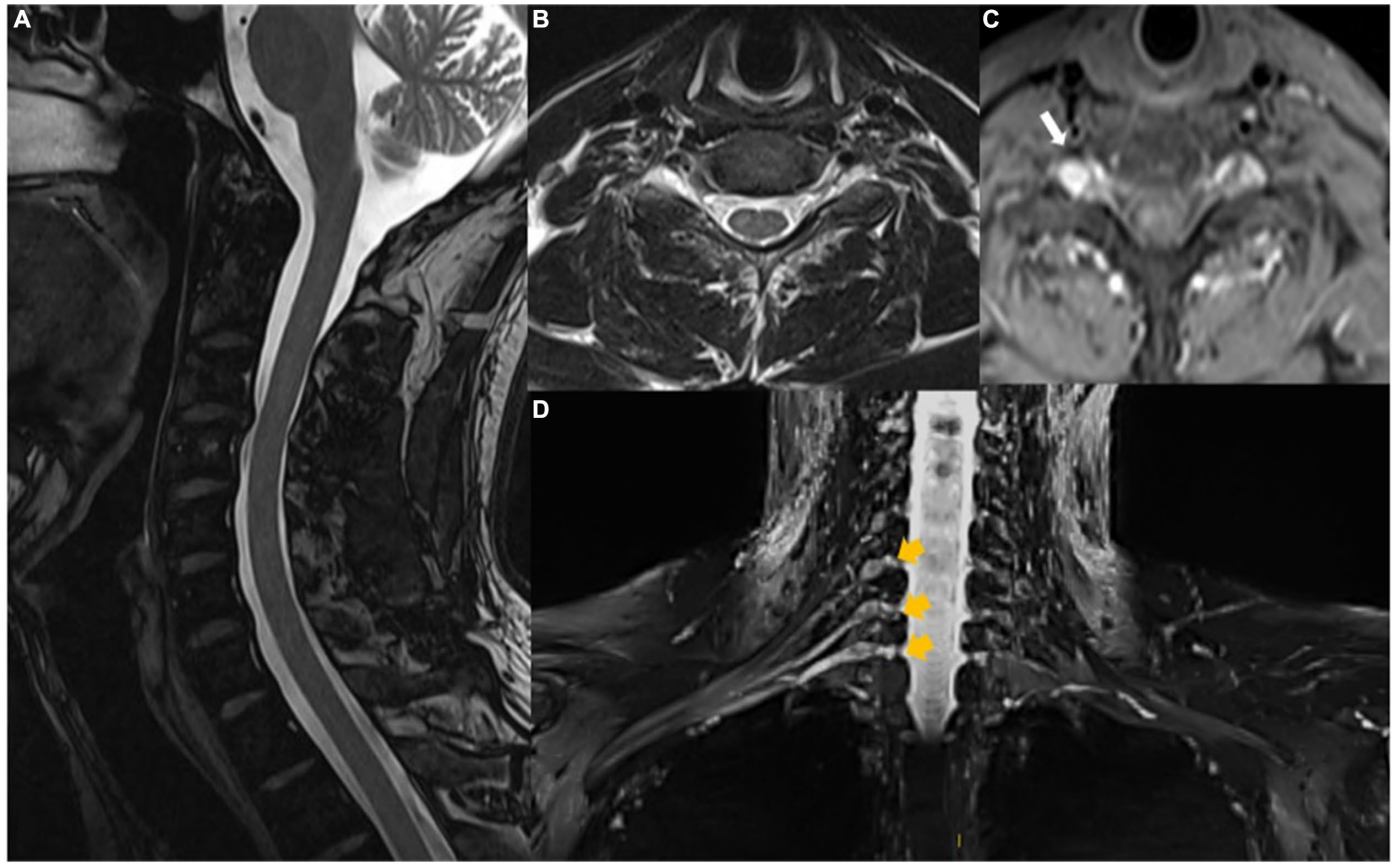
Magnetic resonance of the cervical spine shows that intramedullary changes are no longer observed on T2 **(A,B)** and T1FS after gadolinium **(C)**. Note the reduced size and enhancement of the right root ganglion. **(D)** Coronal STIR MIP with improvement in signal changes and size of cervical roots and brachial plexus on the right (yellow arrows).

Medication for neuropathic pain as monotherapy was given (pregabalin). The patient presented with objective improvement in the pain pattern (allodynia), tactile sensitivity, and strength (MRC grade 4 in the hypothenar muscle). The images also demonstrate significant improvement after 6 months of treatment, as described below: Magnetic resonance neurography of the cervical spine, brachial plexus, right arm, and elbow taken after 6 months of treatment revealed discrete asymmetry in thickness, signal intensity, and contrast enhancement of the neural ganglia of C6, C7, and C8, with only slight edema on the right compared to that observed in the original examination. Thickening, edema, and contrast enhancement of the right neural roots from C6 to T1 of the trunks and cords of the right brachial plexus were evident in the C6 root in the upper trunk and lateral cord. Signs of granulomatous infectious neuropathy and neuritis affecting the nerves of the arm, elbow, and forearm appeared to be resolving after 6 months of treatment. There was a reduction in the intensity of edema and nerve thickening, as well as a decrease in the size of the microabscesses and areas of liquefaction. The signal intensity of edema and thickening of the ulnar nerve along the arm and proximal forearm were also reduced. The nerve, however, remained thickened and enhanced with the contrast medium. There was a marked reduction in the dimensions of the liquefaction at the level of the medial epicondyle. The ulnar nerve in the rest of the forearm had normal thickness and signal intensity in the most recent examinations (Figures 4A–D).

## Discussion

Few reports exist in the literature that describe the involvement of the CNS and proximal nerves, such as ganglionitis and myelitis, in leprosy. Awareness of the more unusual manifestations of leprosy is important for considering and investigating this disease in such cases. This awareness helps ensure that diagnosis and treatment are not delayed.

In this case, the patient did not present skin lesions suggestive of leprosy, which was the first challenge to diagnosis. Previous reports of CNS involvement in leprosy were generally accompanied by classic symptoms of the disease (dermato-neurological), as seen in the case series described by Polavarapu et al., in which six of the eight cases had suggestive skin lesions. In the two cases that did not present lesions, the leprosy diagnosis was performed following a nerve biopsy, as in our case.

Cases of diagnosed multibacillary form leprosy and neuropathy progressing subacutely to myelopathy have been described in the literature (21), as have cases of patients under investigation for neurological conditions who developed erythematous and anesthetic dermatological lesions suggestive of leprosy within a few months (17). Of the cases with CNS involvement reported in the literature, the clinical presentation appears to be varied, ranging from cranial nerve involvement (associated with ataxia) to urinary urgency, changes in the dermatome, or just hyperreflexia, as seen in the patient in this case (18).

We describe the case of a patient who was investigated for neuropathy of the upper limbs, associated with nerve thickening and pain on palpation. However, the initial screening did not point to a classic form of leprosy. After further investigation, several mimicking pathologies were ruled out. The imaging findings drew particular attention, with the involvement of the proximal roots, dorsal root ganglion, and cervical spinal cord in a patient without any symptoms of myelopathy. The absence of signs and symptoms suggestive of ganglionitis and myelitis was noteworthy given that the changes in the images were expressive and the patient presented with involvement of the peripheral nervous system. Such data are generally not seen in leprosy patients, making it a diagnostic challenge. Polavarapu et al. postulated three possible mechanisms for MRI abnormalities in leprosy: (1) retrograde spread of *M. leprae* via the peripheral nerve, plexus, nerve roots, and then to the spinal cord; (2) transection of peripheral nerve axons or nerve roots resulting in changes in the spinal cord; and (3) immunological reaction against the bacterial antigen, which is the most plausible cause of CNS involvement. Jacob et al. (22) suggested that the CNS demyelinating disorder was produced by the phenomenon of molecular mimicry by the cross-reaction of epitope-self-reactive T cells attacking the myelin sheath of nerve axons. Rice et al. suggested that the use of MRI could play an important role during the investigation of patients with such suspicion of suspected proximal injury as it is a sensitive and non-invasive technique for the detection of myelitis and ganglionitis secondary to leprosy, although this requires confirmation in more studies.

Jacob et al. (22) described the case of a young patient with leprosy who developed a demyelinating disorder of the central and peripheral nervous systems and, in addition to being treated with multidrug therapy, the patient was treated with methylprednisolone. The patient responded well and, as in our case, experienced neurological improvement. As postulated by them, the CNS demyelinating disorder was possibly produced by the phenomenon of molecular mimicry by cross-reaction epitopes and autoreactive T cell response that attacks the myelin sheath of nerve axons.

In one study (19), 54 multibacillary patients were assessed, 29 of whom had Wade-Fite stain positivity in a sural nerve biopsy to define leprosy and were then referred to the MRI neuroimaging protocol. Five patients (17.24%) had CNS abnormalities. One patient presented T2/FLAIR hyperintensity of the middle cerebellar peduncle, three had changes suggestive of myelitis and/or ganglionitis, and contrast enhancement in the brachial plexus, dorsal root ganglion, and spinal cord was observed in three patients. As in our case, no characteristics relating to the involvement of the brain or spinal cord were reported for the cases with altered image findings, except in one patient who presented hyperreflexia of the lower limbs, which was also observed in the patient in our case.

The lack of symptoms secondary to the involvement of proximal structures of the nervous system, including the spinal cord, is an interesting feature of this case. It is possible that the signs found were due to edema, which did not cause a mass effect or tissue damage, thus justifying the absence of evident clinical signs of myelopathy.

In our case, we observed an improvement in the imaging findings proximally and distally after the initiation of multidrug therapy as well as a long period of corticosteroid therapy followed by azathioprine as a corticosteroid-sparing agent. Attenuation of the spinal and dorsal root ganglion signal was noted; this is in agreement with another study in which gray-matter spinal cord lesions were completely resolved in two of three cases followed up by MRI (18).

In conclusion, although the majority of leprosy cases present with peripheral neurological findings, we should be aware of the atypical forms of the disease so that the disease is considered and investigated, and patients are diagnosed without delay. In this way, early diagnosis and treatment can minimize the damage and irreversible sequelae of the main form of treatable neuropathy worldwide.

## Data availability statement

The original contributions presented in the study are included in the article/supplementary material, further inquiries can be directed to the corresponding author.

## Ethics statement

Written informed consent was obtained from the individual(s) for the publication office any potentially identifiable images or data included in this article. Written informed consent was obtained from the participant/patient(s) for the publication office this case report.

## Author contributions

CS: Conceptualization, Data curation, Formal analysis, Investigation, Writing – original draft, Writing – review & editing. IP: Data curation, Formal analysis, Investigation, Validation, Writing – original draft. LA: Data curation, Investigation, Writing – original draft. LC: Data curation, Investigation, Writing – original draft. DC: Investigation, Writing – original draft. FS: Investigation, Writing – original draft. ACS: Data curation, Formal analysis, Investigation, Writing – original draft. SM: Data curation, Formal analysis, Investigation, Writing – original draft. ES: Visualization, Writing – original draft. AMS: Investigation, Visualization, Writing – original draft. RP: Supervision, Validation, Visualization, Writing – original draft. MJ: Methodology, Supervision, Validation, Visualization, Writing – review & editing, Writing – original draft.

## References

[1] Ministério Da Saúde. Brasil, Secretaria de Saúde e Desenvolvimento. Atenção Básica. Guia para o Controle da Hanseníase. Brasilia: Ministério daSaúde (2002).

[2] Andrade PR Jardim MR da Silva AC Manhaes PS Antunes SLG Vital R. Inflammatory cytokines are involved in focal demyelination in leprosy neuritis. J Neuropathol Exp Neurol. (2016) 75:272–83. doi: 10.1093/jnen/nlv027, PMID: 26888306

[3] Garbino JA Heise CO Marques WJR. Assessing nerves in leprosy. Clin Dermatol. (2016) 34:51–8. doi: 10.1016/j.clindermatol.2015.10.018 26773623

[4] Jardim MR Vital R Hacker MA Nascimento M Balassiano SL Sarno EN. Leprosy neuropathy evaluated by NCS is independent of the patient’s infectious state. Clin Neurol Neurosurg. (2015) 131:5–10. doi: 10.1016/j.clineuro.2015.01.008, PMID: 25655301

[5] White C Franco-Paredes C. Leprosy in the 21st century. Clin Microbiol Rev. (2015) 28:80–94. doi: 10.1128/CMR.00079-13, PMID: 25567223 PMC4284303

[6] Mowla MR Ara S Mizanur Rahman AFM Tripura SP Paul S. Leprosy reactions in postelimination stage: the Bangladesh experience. J EurAcad Dermatology Venereol. (2017) 31:705–11. doi: 10.1111/jdv.14049, PMID: 27859670

[7] Scollard DM Truman RW Ebenezer GJ. Mechanisms of nerve injury in leprosy. Clin Dermatol. (2015) 33:46–54. doi: 10.1016/j.clindermatol.2014.07.008, PMID: 25432810

[8] Sharma N Koranne RV Mendiratta V Sharma RC. A study of leprosy reactions in a tertiary hospital in Delhi. J Dermatol. (2004) 31:898–903. doi: 10.1111/j.1346-8138.2004.tb00623.x, PMID: 15729862

[9] Van Brakel WH Khawas B. Silent neuropathy in leprosy an epidemiological description. Leprosy Rev. (1994) 65:350–60. doi: 10.5935/0305-7518.19940036, PMID: 7861921

[10] Garbino JA Jardim MR Marques JRW. Hanseníase neural primária. Projeto Diretrizes. Brasília: Associação Médica Brasileira e Conselho Federal de Medicina (2011).

[11] Jardim MR Antunes SLG Santos AR Nascimento OJM Nery JAC Sales AM. Criteria for diagnosis of pure neural leprosy. J Neurol. (2003) 250:806–9. doi: 10.1007/s00415-003-1081-5, PMID: 12883921

[12] Pitta IJR Hacker MA Vital RT Andrade LR Spitz CN Sales AM. Leprosy reactions and neuropathic pain in pure neural leprosy in a reference Center in Rio de Janeiro – Brazil. Front Med. (2022) 9:865485. doi: 10.3389/fmed.2022.865485, PMID: 35402428 PMC8992651

[13] Jenkins D Papp K Jakubovic HR Shiffman N. Leprotic involvement of peripheral nerves in the absence of skin lesions. J Am Acad Dermatol. (1990) 23:1023–6. doi: 10.1016/0190-9622(90)70328-F, PMID: 2172336

[14] Job CK Jayakumar J Williams D Gillis TP. Role of polymerase chain reaction in the diagnosis of early leprosy. Int J Lepr. (1997) 65:461–4. 9465155

[15] Antunes SLG Chimelli L Jardim MR Vital RT Augusto J Corte-Real S. Histopathological examination of nerve samples from pure neural leprosy patients: obtaining maximum information to improve diagnostic efficiency. Histopatologiahansen. (2012) 107:246–53. doi: 10.1590/S0074-02762012000200015 22415265

[16] Khadilkar SV Kasegaonkar PS Ursekar M. Spinal cord involvement and ganglionitis in leprosy. Neurol India. (2007) 55:427–8. doi: 10.4103/0028-3886.33312, PMID: 18040133

[17] Rice CM Oware A Klepsch S Wright B Bhatt N Renowden SA. Leprous ganglionitis and myelitis. Neurol Neuroimmunol Neuroinflamm. (2016) 3:e236. doi: 10.1212/NXI.0000000000000236, PMID: 27218117 PMC4864621

[18] Polavarapu K Preethish-Kumar V Vengalil S Nashi S Lavania M Bhattacharya K. Brain and spinal cord lesions in leprosy: a magnetic resonance imaging-based study. Am J Trop Med Hyg. (2019) 100:921–31. doi: 10.4269/ajtmh.17-0945, PMID: 30761984 PMC6447108

[19] Verma S Garg RK Rizvi I Malhotra HS Kumar N Jain A. Central nervous system, spinal root ganglion and brachial plexus involvement in leprosy: a prospective study. J Cent Nerv Syst Dis. (2022) 14:11795735221135477. doi: 10.1177/11795735221135477, PMID: 36277272 PMC9583215

[20] Medical Research Council. Aids to examination of the peripheral nervous system. London: Her Majesty’s Stationary Office (1976).

[21] Bafna P Sahoo RR Manoj M Wakhlu A. Ganglionitis and myelitis: myriad neurological manifestations of Hansen’s disease. BMJ Case Rep. (2020) 13:e236813. doi: 10.1136/bcr-2020-236813, PMID: 32816936 PMC7437710

[22] Jacob J Alexander M Aaron S Pulimood S Walter N Gnanamuthu C. Acute disseminated encephalomyelitis in Hansen’s disease. Ann Indian Acad Neurol. (2006) 9:166–8. doi: 10.4103/0972-2327.27660

